# FTIR spectroscopy-based lipochemical fingerprints involved in pomegranate response to water stress

**DOI:** 10.1016/j.heliyon.2023.e16687

**Published:** 2023-05-28

**Authors:** Atman Adiba, Rachid Razouk, Abdelmajid Haddioui, Rachida Ouaabou, Anas Hamdani, Mohammed Kouighat, Lahcen Hssaini

**Affiliations:** a National Institute of Agricultural Research, Avenue Ennasr, BP 415 Rabat Principale, 10090, Rabat, Morocco; b Laboratory of Biotechnology and Valorisation of Plant Genetic Resources, University of Sultan Moulay Slimane, BP 523, Beni Mellal, Morocco; c Environmental Technologies, Biotechnology and Valorization of Bio-Resources Team, Abdelmalek Essaadi University, Morocco

**Keywords:** *Punica granatum* L, Seeds oil, Water deficit, Lipochemical traits, Fourier transform infrared spectroscopy

## Abstract

Pomegranate trees are known for their ability to withstand drought conditions, but there is still much to learn about how water stress affects the lipobiochemical behavior of their seeds. This study aimed to investigate how sustained deficit irrigation (SDI-50), equivalent to 50% of crop evapotranspiration, influences pomegranate seed oil attributes such as phenols, flavonoids, and tannins content, and the seeds’ lipochemical fingerprints compared to fully irrigated trees. At the full ripening stage, pomegranate seeds were analyzed for their oil content, biochemical traits, and vibrational fingerprints using infrared radiation. The results indicated that there was a significant genotypic effect coupled with applied water stress on all the investigated traits. Interestingly, an increasing trend in seed oil yield was observed under water stress conditions compared to the control, with the highest oil yield increase observed in the ‘Zheri Precoce’ fruit seeds. Only two cultivars did not show the same pattern, with the oil yield increase ranging from 8% to 100%. Furthermore, SDI-50 induced a substantial increase in total phenolic content, coupled with a significant genotypic effect, and resulted in an average increase of 7.5%. This increase in total phenolics also correlated with an increase in antioxidant activity across all investigated cultivars. ATR-FTIR fingerprinting revealed eleven spectral fingerprints corresponding to functional groups present in pomegranate seeds oil, with a particular pattern of significant effects of both genotypic and SDI-50 factors. These results suggest that exploiting water scarcity conditions could be a viable approach to improve the quantitative and qualitative attributes of pomegranate seed oil. While there are still several aspects to be investigated further, this study provides a basis for pomegranate processing under water shortage conditions.

## 1 Introduction

Pomegranate (Punica granatum L.) is a well-known fruit in the Mediterranean region that contains a variety of health-promoting compounds, making it an important component of the Mediterranean diet [1]. Its medicinal properties and ecological role have also gained particular interest [1]. The edible portion of the fruit, the arils, accounts for approximately 50–70% of the total fruit and consists of about 60–85% juice and 15–22% seeds and 33–40% peels [2].

Due to the increase in pomegranate production, there has been an increase in the annual production of pomegranate seeds, which are often disposed of as waste products in many pomegranate-processing industries. This represents 3–6 million tons per year [[3], [4], [5]]. Although no data on pomegranate seed production are available for Morocco, which is considered one of the main African pomegranate fruit producers, it is known that the growing interest in pomegranate fruits is related to their demand for industrial processing to obtain pomegranate juice, oil, and jams, and their use in traditional medicine due to their beneficial properties in the human diet, such as the reduction of the risk of chronic disorders, including atherosclerosis, cancer, diabetes, and other diseases [6].

Pomegranate seeds have been reported to have an important oil yield (12–20%) and to present high benefits due to their unique nutraceutical and bioactive characteristics [7]. Pomegranate seed oil is rich in polyphenols, such as flavonoids, tannins, anthocyanin, hydroxybenzoic acids, and hydroxycinnamic acids, and in lipidic composition, such as fatty acids, sterols, tocopherols, and phospholipids [8,9]. These components have been reported to inhibit β-catenin, epithelial-mesenchymal transition expression, and metastasis in triple negative breast cancer cells [10], to reduce chemotherapy-induced liver damage [11], and to maintain the balance and stability of food's physicochemical properties and their nutrient profile [12,13].

In many countries, oil-bearing seeds, such as pomegranate seeds, have driven the emergence of impactful businesses in line with the circular economy concept [14]. However, climate change is becoming a major challenge for agriculture in many regions, including Morocco, where drought is one of the main changes threatening natural resources and consequently agriculture [15]. Climate predictions have indicated that the increased aridity in Morocco will continue, with an additional increase in mean temperature of 1.5 °C by 2050 under the climate change scenario RCP4.5. This will be associated with a decrease in rainfall of about 15% [16]. Pomegranate, although known as a resilient tree, has been shown to be severely impacted by water stress under Moroccan weather conditions [[17], [18], [19]]. This impact has been measured through several molecular and physiological fingerprints over both the tree and the fruit. However, studies on the effect of water stress on pomegranate seed oil are limited, and no available research has assessed this influence over lipochemical attributes of pomegranate seeds and how drought can shape the available opportunities to shift to a circular economy model in the pomegranate processing pathway. The current study aims to investigate the impact of water stress on pomegranate seed oil content and their lipochemical properties. Specifically, this research will examine the effects of two irrigation regimes (50% ETc and 100% ETc) on the oil yield and lipochemical properties of pomegranate seeds from seven different cultivars grown in an ex-situ collection. To assess these properties, Fourier transform infrared spectroscopy (FTIR) will be used to compare the lipochemical profiles of seeds grown under different irrigation regimes. This study will not only provide novel insights into the effects of water stress on pomegranate seed oil, but also explore the potential of integrating seeds’ lipo-biochemical attributes into investigating drought-resilience plasticity in pomegranate plants. This study makes a significant contribution to the field by being the first to investigate the effect of sustained deficit irrigation on the lipobiochemical behavior of pomegranate seeds. The findings provide novel insights into the underlying mechanisms of pomegranate’s drought tolerance and reveal the potential to enhance the quantitative and qualitative attributes of pomegranate seed oil under water scarcity conditions. Additionally, this research could open up new avenues for developing sustainable agricultural practices in arid regions.

## 2 Materials and methods

### 2.1 Experimental site

**Fig. 1:**
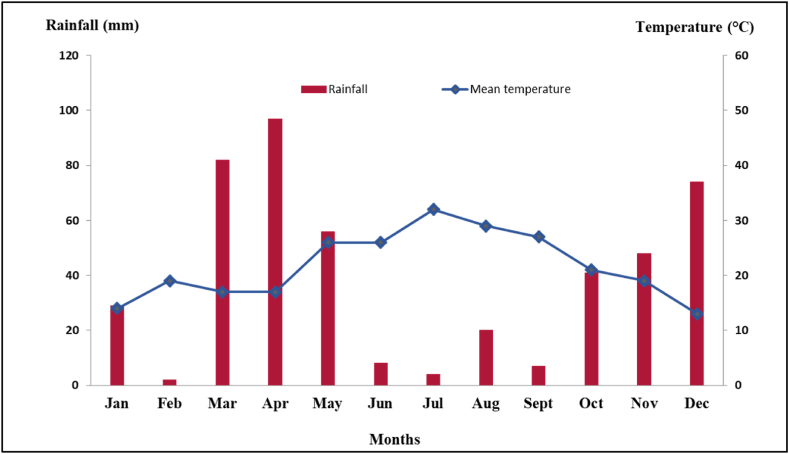
Monthly rainfall and mean temperature in the experimental orchard in 2020 recorded from an in-situ weather station.

This study was carried out at the experimental station of the National Institute of Agricultural Research (INRA) of Meknes-Morocco in the Sais Plain (latitude 33°56′N, longitude 5°13′O, altitude 499 m), characterized with the semi-arid Mediterranean climate known with hot and dry summers. The annual reference evapotranspiration (ET_0_) was 1067 mm and the total annual rainfall was 250 mm recorded during 2020 from an in-situ automated weather station. The monthly distribution of rainfall and temperature is typically irregular showing that the rainfall deficit was more pronounced between March and August (Fig. 1). The average value of ET_0_ was 775 mm during the active growth season of pomegranate (April–October).

The soil of the experimental plot was a sandy-clay texture, moderately alkaline (pH 7.68), slightly calcareous (3.05% CaCO_3_), moderately rich in organic matter (2.04%), phosphorus and potassium, with a useable water reserve of 1.7 mm cm^−1^.

### 2.2 Pomegranate orchard and irrigation treatments details

Seven pomegranate cultivars of thirteen-year-old were involved in this study, five of which were local clones, namely ‘Grenade Jaune’, ‘Djebali’, ‘Ounk Hmam’, ‘Sefri’, and ‘Gjeibi’ and two were exotic varieties: ‘Zheri Precoce’ (Tunisia) and ‘Mollar Osin Hueso’ (China). Trees were planted at 5 × 3 m in a complete randomized design. The growing season of the aforementioned cultivars begins in early April till the end of October. Water stress was applied during the fruit set stage occurring in early May. Hence, two irrigation treatments were applied using one lateral pipe parallel to the tree row and two emitters per tree each delivering 8 or 16 L h^−1^ depending on the treatment. The first treatment is a control, in which the trees were irrigated at 100% to guarantee -non-limiting soil water conditions. The second treatment was a sustained deficit irrigation treatment (SDI-50), in which 50% of crop water requirement (severe stress) was constantly applied.

Crop water requirements were scheduled according to daily ET_0_ and the crop coefficients (Kc) obtained by Doorenbos and Pruitt [20]. The Kc values were adjusted to tree canopy cover (Sc) using the reduction coefficient (Kr) expressed as Eq. (1), where N is the planting density and D is the average canopy cover diameters [21].

(1) $$Kr=\frac{2.Sc}{100}\;with\;Sc=\frac{\pi \cdot D^{2}\cdot N}{100}$$

To avoid interactions regarding soil water between adjacent trees of different cultivars, each water treatment was applied on a separate line containing one examined cultivar with five replicates (trees) per irrigation treatment. However, only the central trees of each cultivar per treatment were used for measurements and the other trees acted as buffer plants.

### 2.3 Measurements

At their full ripening stage, 20 pomegranate fruits were randomly harvested from each tree (60 per cultivar) under each water regime. Their arils were separately and manually turned into juice. Shortly afterward, seeds were collected as residue and washed with excess water for the removal of sugars and any adhering materials and then left for 24h to dry at room temperature. Later, the seeds were crushed and sieved to obtain a fine powder.

#### 2.3.1 Oil content determination

Pomegranate seed oil (PSO) content was measured using Nuclear Magnetic Resonance (OXFORD 4000 NMR Analyzer) calibrated against 30 mL of previously extracted oil and its respective dried cake (0% of oil), early weighted. For each sample, PSO content was measured by placing seeds fine powder into a special glass tube which was introduced into the NMR device to calculate PSO content. This analysis was performed following eight replicates for each sample. The value displayed by the device expresses the oil content of pomegranate seeds dry weight. The oil content was measured according to Eq. (2), where OCF is the oil content of fresh weight, OCD is oil content of dry weight and H is the seeds water content.

(2) OCF = OCD × (100 − H)/100

#### 2.3.2 Oil extraction

For each pomegranate cultivar, 30 g of the seeds powder of a randomly chosen cultivar was used to extract oil using hexane (200 mL) as a solvent. Extraction was performed at 30 °C for 8 h followed by solvent evaporation using a rotary evaporator (Büchi R-205, Switzerland) under a speed of 1500 rpm and reduced pressure, at 40 °C. The powder obtained (0.5 g) of each sample of pomegranate seeds was extracted with 30 mL of 80% methanol. After a continuous shaking at 4 °C for 20 min on an orbital shaker, the sample extract was subsequently filtered using the Whatman No. 1 filter paper. The combined extracts were kept in dark at −20 °C until analysis. The methanolic extract serves for the determination of total phenolic content, tannins, antioxidant activity and total flavonoid content.

#### 2.3.3 Determination of phenolic compounds

Total phenolic content was measured according to the Folin–Ciocalteu method described by Singleton et al. [22], with some modifications. Hence, an aliquot of 0.5 mL of extract and 1.5 mL of Folin–Ciocalteure agent diluted 1/10 were mixed. After 3 min of incubation at room temperature, 1.2 mL of sodium carbonate (75 g/L) was added and the mixture was incubated in the darkroom at temperature for 90 min.

The absorbance was measured at 750 nm using a spectrophotometer (UV-1700 Shimadzu, Japan), with gallic acid as a standard within a range of concentrations from 0 to 1000 mg/ml in increments of 100 mg/mL. The total phenolic content was expressed on a dry weight basis as mg gallic acid equivalents/100 g of sample (mg GAE/100 mL). The equation obtained from the standard curve, y = ax + b with an R^2^ value of 0.99, was used to determine the total phenolic content of the samples.

#### 2.3.4 Determination of flavonoids content

PSO flavonoid content was measured according to the colorimetric method developed by Bouaziz et al. [23] with slight modifications. Hence, 1 mL of PSO methanolic extracts was mixed with 4 mL of distiller water. Then 0.3 mL of NaNO_2_ (5%) was added. After 5 min of incubation at room temperature, 0.3 ml of aluminum trichloride (10%) was added, and then 6 min later, 2 mL of NaOH solution (1 M) and 2.4 mL of distilled water were added. After vigorous shaking, the absorbance was read at 510 nm using a spectrophotometer (UV-1700 Shimadzu, Japan). Total flavonoids were quantified using quercetin by dissolving 5 mg quercetin in 1.0 mL methanol, then serial dilutions were prepared using the same solvent (0, 20, 50, 100, 200, 300, 400, 500 and 600 μg/mL) and their absorbance was measured following the same procedure above cited. Results were expressed on a dry weight basis as mg quercetin equivalents (QE)/100 g of sample. The linear equation y = ax + b, which was derived from the standard curve with an R^2^ value of 0.99, was employed to quantify the total phenolic content of the samples.

#### 2.3.5 Determination of tannins content

The estimation of total tannins content of pomegranate seed oil was assessed according to the method of Willis, [24]. Briefly, 1 mL of such extract and 2 mL of methanol (4%) were mixed. Then, 750 μL of HCl was added. The solution was incubated at room temperature for 1 h. After vigorous shaking, the absorbance was read at 550 nm on a spectrophotometer (UV-1700 Shimadzu, Japan). The water was used as a blank. Then, the standard solutions were prepared from diverse concentrations of tannic acid solutions (0, 100, 200, 500, 1000, 1500 and 2000 mg/L). The total tannins content was presented as mg tannic acid equivalent (TAE) per mL of oil and determined using the equation y = ax + b, which was derived from the standard curve and had an R^2^ value of 0.99.

#### 2.3.6 Determination of DPPH free radical scavenging activity

PSO capacity to scavenge 2,2-diphenyl-1-picrylhydrazyl (DPPH) free radicals was measured according to the method described by Siano et al. [25]. Hence, 0.25 ml of PSO phenolic fraction of each sample was mixed with 0.5 ml of a methanolic solution (80%) containing DPPH radicals (6 × 10^−6^ M). The mixture was vortexed vigorously and incubated for 30 min in the dark at room temperature, and then absorbance was measured at 517 nm. The absorbance of DPPH in 80% methanol was measured as a control. The result of the DPPH scavenging effect was calculated as the percentage of DPPH discoloration using the following equation:

(3) % scavenging effect = [(Abs_control_ − Abs_sample_)/Abs_sample_] × 100

#### 2.3.7 FTIR spectroscopy

Vibrational fingerprints of PSO were acquired in the mid-infrared region (4000–500 cm^−1^) using a Vertex 70-RAM II Bruker spectrometer equipped with a diamond attenuated total reflectance (ATR) accessory at a resolution level of 4 cm^−1^, averaging 256 scans per spectrum. Infrared (IR) spectra were acquired by applying 50 μL of each PSO sample on the ATR cell in the room conditions; the IR of each sample corresponded to an average of ten replicates. IR spectrum of empty germanium crystal surface was set as background, which was automatically subtracted from IR spectra of each PSO sample. Between each IR acquisition, ethyl alcohol diluted with warm deionized water was employed with the use of soft paper to clean the ATR cell.

### 2.4 Statistical analysis

The statistical analysis involved investigating the effects of two study design factors: water regime applied and cultivars. Normality and homogeneity tests were conducted on the data using SPSS v27. Multiple analysis of variance (MANOVA) was then performed to determine the magnitude of the effect of these factors and their interactions, followed by a Duncan assay to compare means. To prepare the FTIR data, standard normal variate (SNV) and multiplicative scattering correction (MSC) procedures were employed. The resulting corrected IR spectrum and integrated intensities were plotted using Originlab pro software. Next, the FTIR and biochemical data were analyzed separately using principal component analysis (PCA) to identify the variables and spectral signatures that contributed the most to explaining the water stress effect on PSO attributes. Finally, two-dimensional scatter plots were generated to visualize the classification pattern of sampled cultivars induced by the aforementioned effects.

## 3 Results

### 3.1 Effect of SDI on seed oil yield

Water stress effects on the seed oil yield of the seven pomegranate cultivars are shown in Table 1. PSO yield was significantly increased by SDI-50 treatment in five cultivars compared to control fruit seeds. The highest increase was recorded in the ‘Zheri Precoce’ cultivar, where the oil yield level was twice that of the control, followed by ‘Grenade Jaune’, with an increased ratio of 75%. However, the lowest increase of seed oil was observed in the ‘Sefri’ cultivar with a ratio of 8.33%. ‘Gjeibi’ and ‘Mollar Osin Hueso’, both showed a similar rate of oil yield increase as a response to the water stress compared to the control (66.67%). On the other hand, the oil yield decrease pattern caused by water stress was not as dramatic as that of the increase. Hence, the decrease in oil yield was specifically observed in the ‘Ounk Hmam’ and ‘Djebali’ cultivars of which the recorded ratio was 16.67% and 37.5%, respectively.

**Table 1:** Seeds oil content (%) of seven pomegranate cultivars under SDI-50 and the control treatment.

Cultivar	Seeds oil content (SOC) (%) under 100% ETc	Seeds oil content (SOC) (%) under SDI-50	Impact (%)
Sefri	12	13	+8.33
Djebali	8	5	−37.5
Mollar Osin Hueso	6	10	+66.67
Gjeibi	6	10	+66.67
Ounk Hmam	6	5	−16.67
Zheri Precoce	4	8	+100
Grenade Jaune	4	7	+75

Impact ratio (IR) was calculated as follows: $IR=\frac{SOC\left(SDI-50\right)-SOC\left(100\%ETc\right)}{SOC\left(100\%ETc\right)}*100$.

### 3.2 Biochemical proprieties

**Fig. 2:**
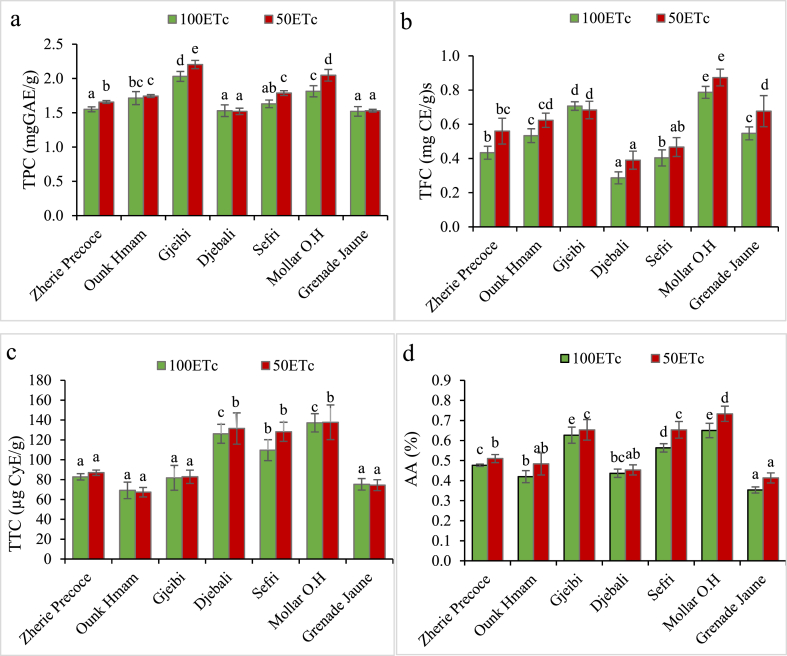
Effect of different irrigation treatments (Control and SDI-50) on total phenols content **(A)** (TPC, g GAE/L), flavonoids **(B)** (TFC, mg CE/g), tannins **(C)** (TTC, μg CyE/g) and antioxidant activity **(D)** (AA, %) in seven-pomegranate seeds oil. Duncan test was used for mean comparison.

Sustained deficit irrigation treatment did not affect the seed oil total phenolic content in ‘Djebali’ and ‘Grenade Jaune’ cultivars (Fig. 2a). However, a significant increase in PSO TPC was observed in ‘Zheri Precoce’, ‘Ounk Hmam’, ‘Gjeibi’, ‘Sefri’ and ‘Mollar Osin Hueso’ cultivars under SDI-50 treatment according to the following rates of 6%, 1.8%, 4%, 9% and 11%, respectively. PSO of the stressed fruits showed the highest total flavonoid levels in comparison to the full irrigated fruits in all sampled cultivars except ‘Gjeibi’, ‘Djebali’ and ‘Mollar Osin Hueso’ (Fig. 2b). On the other hand, a high increase of total tannins content was observed in seeds oil of ‘Mollar Osin Hueso’ and ‘Djebali’ with average rates of 0.3%, 4% and 17%, respectively. However, no significant difference was revealed between the deficit irrigation and full irrigation treatments on PSO TTC content (Fig. 2c). Regarding the free radical scavenging activity based on the DPPH assay, results from stressed samples showed high scavenging capacity in comparison to that of seed oil from fruit grown under 100% ETc (Fig. 2d). The greatest increase in antioxidant activity (16%) was observed in the cultivar ‘Sefri’ and ‘Ounk Hmam’ (15%), followed by the cultivars ‘Zheri Precoce’, ‘Djebali’, ‘Mollar Osin Hueso’ and ‘Gjeibi’ having an average of 6.33%.

### 3.3 FTIR profiling

**Fig. 3:**
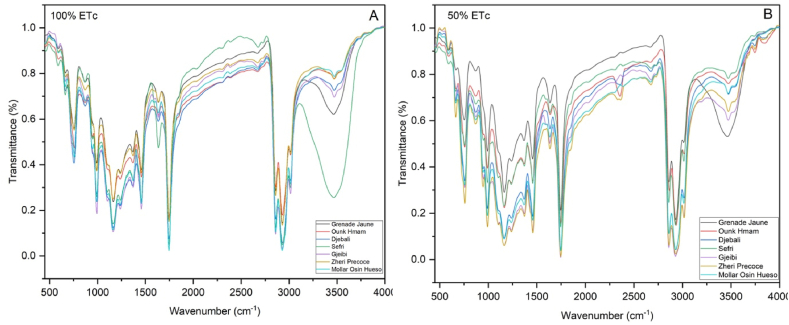
Averaged FTIR spectra of seven pomegranate cultivars under two irrigation treatments - full irrigation (control) (A) and sustained deficit irrigation (SDI-50) (B) - in the mid-infrared region (4000-500 cm-1). Each infrared spectrum represents the average of ten replicates, with each replicate obtained by accumulating 128 scans.

**Fig. 4:**
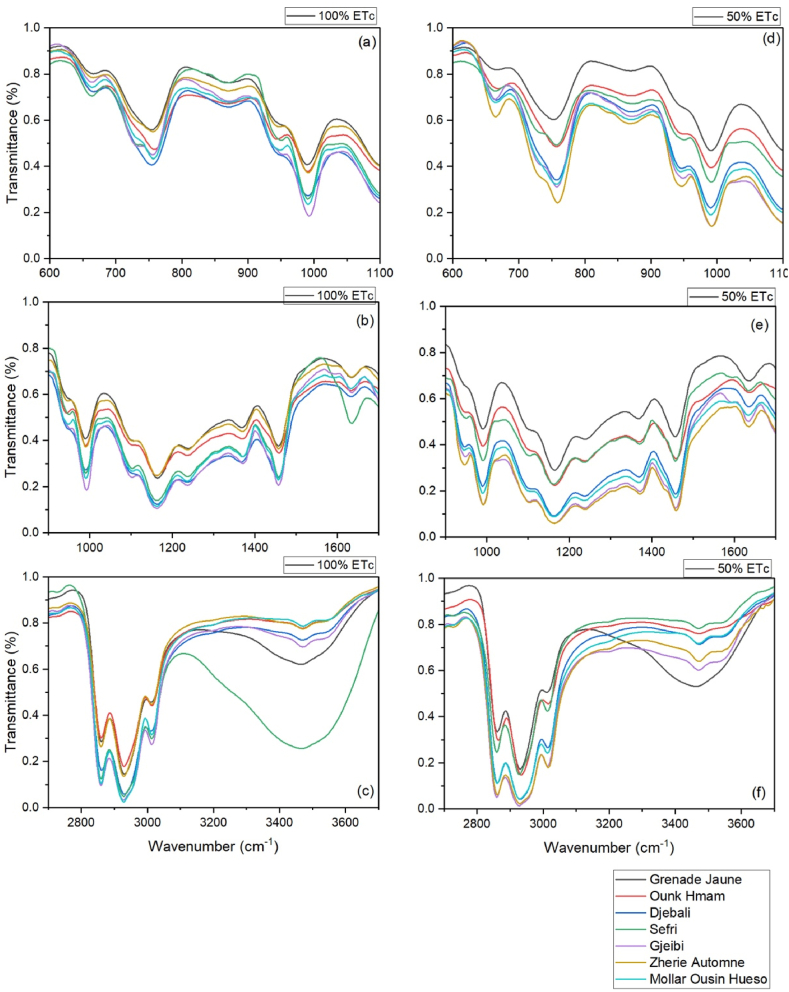
FTIR major vibrational regions under control and deficit irrigation conditions, illustrating the genotypic effect. Panels (a), (b), and (c) represent the main vibrational region under full irrigation, while panels (d), (e), and (f) correspond to the same vibrational region under sustained deficit irrigation (SDI-50).

**Fig. 5:**
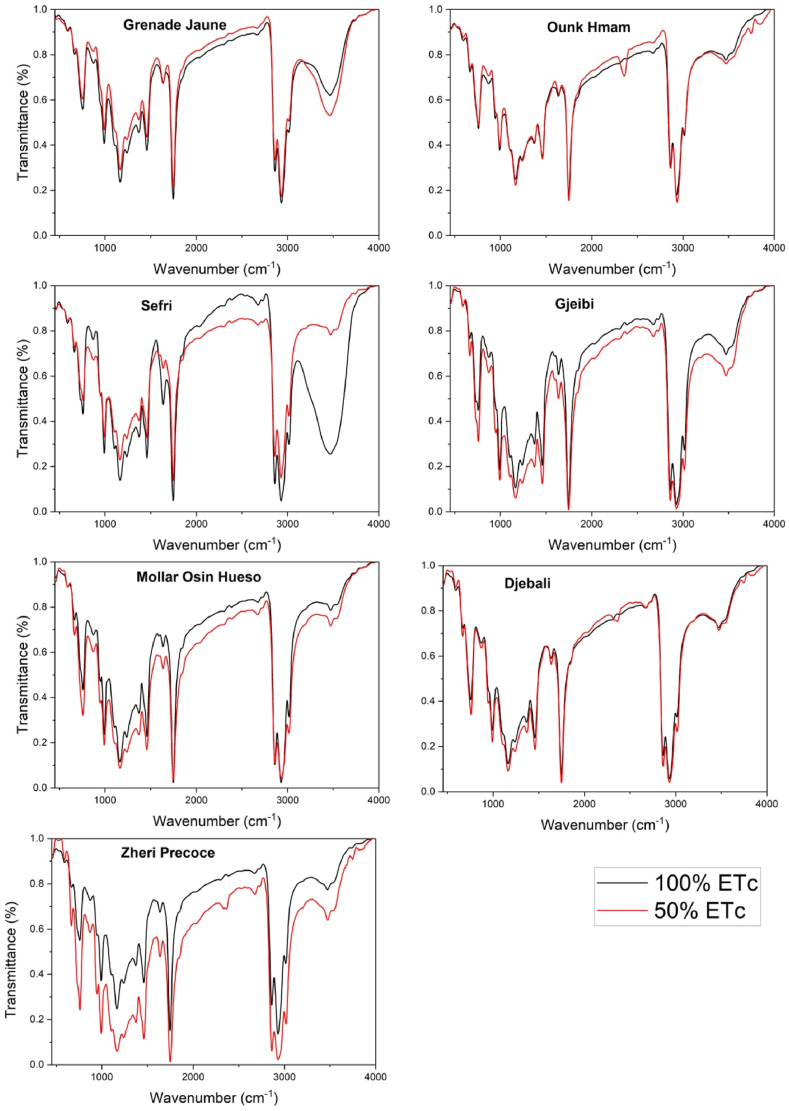
FTIR spectra of pomegranate seed oil showcasing the genotypic response to SDI-50 and control conditions. Each panel represents one of the investigated samples under both treatments, allowing for direct comparison between the two.

The FTIR spectra profile of PSO lipochemical fingerprints for the seven pomegranate cultivars conducted under full irrigation and water stress treatments were illustrated in Fig. 3, Fig. 4, Fig. 5. Assignments of FTIR peaks to their respective specific functional groups were given in Table 2 based on the works of De la Mata et al. [26], Hssaini et al. [27], Movasaghi et al. [28], Chandra et al. [29], Niu et al. [30] and Hssaini et al. [31]. Results showed 11 peaks identified as PSO molecular fingerprints of which the vibrational intensities corresponded to the varietal factor effect combined with the response to water stress.

**Table 2:** FTIR band assignments of pomegranate seed oil.

Spectral range	Band assignments
3471	NH stretch, coupled with HB
3013	m(C-H) of cis-alkene (-HC**-**CH-)
2927	m(C-H) from methylene (-CH_2_) groups of lipids
2859	Terminal (-CH_3_) groups
1745	(C-O) stretching vibration of carbonyl groups belonging to the triacyglycerols
1635	Amide I: β-sheet
1457	(CH_2_) bending and deformation of methylene of lipids, proteins, or cholesterol esters
1372	(CH_3_) bending vibration (lipids and proteins)
1163	Amide III band region
991	Amide III: β-sheet
757	(CH) bending

Assignments were made based on the following references: De la Mata et al. [26], Hssaini et al. [27], Movasaghi et al. [28], Chandra et al. [29], Niu et al. [30] and Hssaini et al. [31].

The absorption band at 3471 cm^−1^ was ascribed to NH stretch, coupled with HB. At this wavenumber, the cultivar ‘Sefri’ typically showed a high vibrational intensity under control conditions; though, this absorbance intensity was the lowest under water stress.

The stretching vibration band around 2927 cm^−1^ was assigned to C-H of methylene and -CH_2_ groups of lipids. At 2859 cm^−1^ a sharp peak was identified with the highest absorbance most often attributed to the region of the terminal -CH_3_ groups. More interesting, the transmittance becomes very prominent within low-wavenumber regions with a remarkably strong absorption band at 1635 cm^−1^ characteristic of the amide I: β-sheet, as well as bands at 991 cm^−1^ and 757 cm^−1^ which are characteristic of amide III: β-sheet and (CH) bending respectively.

A remarkable difference was observed between the FTIR spectra of lipobiochemical fingerprints of seeds oil between the fruits growing under 50% ETc and 100% ETc regimes (Fig. 3, Fig. 4, Fig. 5). The results were reported that the FTIR spectra of the seeds oil of 50% ETc and 100% ETc of the ‘Grenade Jaune’, ‘Ounk Hmam’ and ‘Djebali’ cultivars are confused, which indicated that the unceasing irrigation water quantity had no effect on the seeds lipobiochemical proprieties of this cultivars. However, for the other cultivars (‘Gjeibi’, ‘Zheri Precoce’, ‘Moller Osin Hueso’ and ‘Sefri’) high transmittance intensity was observed in the seeds extracted from the control fruits in comparison with the seed oil of the fruits growing under SDI-50. In the ‘Sefri’ cultivar, the data analysis reported the appearance of a specific spectral peak only in the seeds oil control around the vibrational region of 3400–4500 cm^−1^.

## 4 Discussion

### 4.1 Seed oil yield

To the best of our knowledge, there is no previous report regarding the effect of deficit irrigation on pomegranate seed oil yield and its biochemical attributes. The findings herein reported proved the pomegranate tree plasticity to water deficit. Hence, a significant effect of sustained deficit irrigation on pomegranate seed oil yield was observed in all studied cultivars. Oil yield under control treatment has ranged between 4 and 10%. Similar yield levels were given by Fernandes et al. [32] who reported that the seed oil yield examined for nine pomegranate cultivars grown in Spain ranged between 4.44 and 13.7%. These levels are nevertheless, lower than those reported by Fadavi et al. [33] over twenty-five Iranian cultivars (6.63–19.3%) and Loukhmas et al. [34] who reported oil yield levels between 17.59 and 24.69% regarding ten pomegranate cultivars growing in Morocco. Likewise, higher yield levels (4.9–26.8%) were reported in the work conducted in Italy by Ferrara et al. [35]. The differences above cited are most often due to genotypic factors as well as environmental conditions [34]. On the other hand, in comparison with the other species, a level quite similar to the pomegranate seed oil content reported for Prickly pear (5.3–7.7%) [36,37], date palm (5.0–9.7%) [38,39] and strawberry (6.2%) [40].

In this research, samples conducted under SDI-50 treatment showed a substantial increase in their seeds oil yield which might probably be explained by the increase of the seed/aril ratio during the fruit set which is likely a physiological response to water deficit [41,42]. However, several other researches on the other species reported that water stress induced a significant increase in the oil yield without any effect on the seed yield which can be explained by the fact that water stress activates the oil biosynthesis process of seeds, such as sunflower [43,44], rapeseed [45], sesame [46], oil seed rape [47], black cumin [48], camelina and dragon's head [[49], [50], [51]].

### 4.2 Oil biochemical attributes

PSO biochemical attributes displayed various patterns as a response to the cultivar factor, water stress and their interaction. This response was statistically significant among cultivars, except for tannins, while water stress seemed not having a significant effect on these attributes. The response to the interaction between both above-cited factors seemed not having any effect on PSO biochemical traits. Phenolic compounds are directly involved in the prevention of oxidation and oil preservation. The levels revealed by cultivars herein tested were higher than those reported by Amri et al. [52] and Schubert et al. [53] where the average values were 93.42 mg/kg and 150 mg/kg respectively. In comparison to the control treatment, a significant increase in TPC was observed under water stress in five pomegranates involved in the present study. Similar trends were reported on Maize [54], olive [55,56] and rapeseed oil [57]. These results can be explained by the fact that water stress induced the activation of the phenylalanine ammonia-lyase, which is considered the key enzyme in phenolic compounds biosynthetic pathway and the responsible of phenols accumulation [58]. For flavonoids content, similar values were reported by Jing et al. [59] over four Chinese pomegranate cultivars (0.37–0.58 mg CE/g), and Amri et al. [52] who reported a total flavonoids content of 0.594 mg/g in seeds oil of pomegranate cultivar grown in Tunisia. However, higher levels were reported by Zhang et al. [60] who reported a range between 81.9 and 114.7 mg RE/g.

Flavonoids are secondary metabolic compounds playing an important physiological role in plant stress tolerance. Several studies reported the increasing trend of seeds oil flavonoid content under deficit water conditions in several plant species [61,62]. A similar pattern was herein observed only for ‘Sefri’, ‘Zheri Precoce’, ‘Ounk Hmam’ and ‘Grenade Jaune’ cultivars with an increasing rate ranging between 8 and 32%. These results suggested that the variation of the flavonoid content of seeds oil of pomegranate fruits under water deficit might be cultivar-dependent.

Tannins are a group of phenols compounds synthesized through vegetal secondary metabolism. A significant increase in these compounds was observed in three pomegranate cultivars herein examined with an average rate in the range of 0.3 and 17%. Similar researches have reported a significant increase of up to 14% in tannins content induced by moderate water stress, while severe water stress has been reported to induce an increase of up to 91% compared to full irrigation [63].

The free radical scavenging varied within a narrowed interval of 0.35 mg/ml and 0.65 mg/mL. This range is comparable to that reported on Tunisian cultivars by Amri et al. [52] on *cv Tounsi* (0.37 mg/ml) and Khemakhem et al. [64] (0.10 and 0.30 mg/mL). Likewise, previous studies on cultivars growing in Morocco by Loukhmas et al. [34] and Brazilian Melo et al. [65] reported an average antioxidant activity of 1.80 mg/L and 3.77 mg/L, respectively. These differences are probably due to genetic variation and growing conditions [34]. According to Elfalleh et al. [66] a significant effect of the extraction method has been reported on oil free radical scavenging activity. The high antioxidant activity in pomegranate seed oil supports the research that calls for the use of pomegranate seed oil as a functional food and the formulation of nutraceuticals for human health [67]. Khemakhem et al. [64] indicated that pomegranate seed oil has been tested for its ability to prevent diseases associated with oxidative stress such as cancer, cardiovascular and neurodegenerative diseases. The same conclusions were reported for the pomegranate juice, with a significant positive effect on kidney, liver, heart and testis histopathological changes, the tissues lipid peroxidation and body antioxidant status [68]. However, the study conducted by Zahedi et al. [69] reported that the antioxidant activity of pomegranates is influenced by various environmental conditions, including drought stress and tree level status. These findings suggest that the growth conditions of the pomegranate trees can impact the antioxidant properties of the fruit. Specifically, the study highlights the importance of considering environmental factors when studying the antioxidant activity of pomegranates. Overall, the results of this research contribute to our understanding of the complex relationship between environmental conditions and the health benefits of consuming pomegranates.

In our study, a significant increase in antioxidant activity under sustained deficit irrigation was observed in five cultivars. The Reports on the effect of the water stress on the pomegranate seeds oil were still very limited. But several researches have reported an increase in the antioxidant activity under water deficit in pomegranate juice which may be due to the increase of antioxidant compounds as previously described [70,71]. Thus, Zahedi et al. [72] reported a significant increase in the antioxidant activity of the pomegranate leaves of three commercial Iranian cultivars under intense drought stress.

### 4.3 FTIR spectroscopy

One of the main objectives of this part was the assessment of the effect of the applied water stress on the lipochemical fingerprints of seven pomegranate cultivars using the Fourier Transform Infrared (FTIR) spectroscopy. Indeed, in combination with chemometric techniques, this method has been successfully used for the detection of adulteration of fruit purees, honey, and oils of many species. For the pomegranate fruits, FTIR spectroscopy was generally used for the study of the authentication of pomegranate juice concentrate [73], detection of food fraud in commercial pomegranate molasses syrups [74] and the assess microbial quality of minimally processed pomegranate arils [75].

For the seeds oil analyses, several species were tested using FTIR spectroscopy such as fig [76], olive [77] and sunflower [78]. On the other hand, for the pomegranate seed oil, the use of the FTIR spectroscopy was generally based on the test of the effect of the extraction method on the quality of the pomegranate seed oil [79] or the test of the capacity of the pomegranate seed oil to preserve the food quality [80].

### 4.4 Interactions between factors

Table 3 summarized the MANOVA results regarding the impact of the experimental design on PSO lipobiochemical. Overall, the experimental design factors (cultivar and water regime) had separately a statistically significant effect on PSO quality. Though, their interaction seemed not to have a significant effect, which could be read through to the Wilks’ Lambda value recorded for water stress (λ = 0.577) as being the highest compared to that of the cultivar factor (Table 3). Looking at the test of between-subject effects, it was revealed that only free radical scavenging activity (AA) and total flavonoid content (TFC) showed significant effects at *p* < 0.001 and *p* < 0.05, respectively, under the interaction between the design factors. Individually, the design factors had a statically significant impact on all herein studied variables except for total tannins content, which did not display a significant difference among sampled cultivars. Partially, similar results were reported on the same cultivars regarding their whole fruits’ response to water stress over two consecutive years [18]. Referring to the same study, the interaction cultivars*water regime displayed the highest value of Wilk’s lambda, meaning the lowest effect on the pomegranate biochemical attributes. These results are interesting as they describe the effect of each factor on the herein investigated parameters and how this magnitude was affected based on the interaction levels.

**Table 3:** Multiple analyses of variance (MANOVA) displaying the impact of experimental design factors and their interaction on PSO lipobiochemical attributes.

Multivariate tests^a^					
Effect	Wilks' Lambda value	F	Hypothesis df	Error df	Sig.
Intercept	.001	5.350.361	4.000	25.000	.000
Cultivar (C)	.001	20.393	24.000	88.425	.000
Water regime (WR)	.577	4.586b	4.000	25.000	.006
C * WR	.509	.787	24.000	88.425	.744
Tests of between-subjects effects					
Source	Dependent Variable	Df	Mean Square	F	Sig.
Intercept	TPC	1	126.187	22.913.273	.000
	TTC	1	417.226.594	2.910.405	.000
	TFC	1	14.597	495.600	.000
	AA	1	11.724	9.907.366	.000
C	TPC	6	.252	45.696	.000
	TTC	6	4.955.713	34.569	.000
	TFC	6	.149	5.067	.001
	AA	6	.094	79.080	.000
WR	TPC	1	.070	12.790	.001
	TTC	1	212.130	1.480	.234
	TFC	1	.135	4.579	.041
	AA	1	.016	13.861	.001
C * WR	TPC	6	.007	1.361	.265
	TTC	6	69.852	.487	.812
	TFC	6	.031	1.059	.410
	AA	6	.001	.786	.588

ns: non-significant difference; ∗: significant difference at p < 0.05; ∗∗: significant difference at p < 0.01.

C: Cultivar; WR: Water regime.

TPC: Total phenolic content; TTC: Total tannins content; TFC: Total flavonoids content; AA: antioxidant activity.

a Design: Intercept + C + WR + C * WR.

The mechanisms of the pomegranate oil yield increasing, biochemical proprieties and lipochemical fingerprints variation under water stress regime were reported in Fig. 7. Thus, water stress can induce a range of physiological responses in plants, including changes in gene expression, metabolism, and water use efficiency. In the case of pomegranate plants, water stress appears to particularly stimulate the production of oil in the seeds, potentially as a response to the environmental stress. The main mechanisms underlying this increase in oil yield are not yet fully understood, but some research suggests that water stress can alter the activity of enzymes involved in fatty acid synthesis and oil accumulation, leading to increased oil production in the seeds [81,82].

**Fig. 6:**
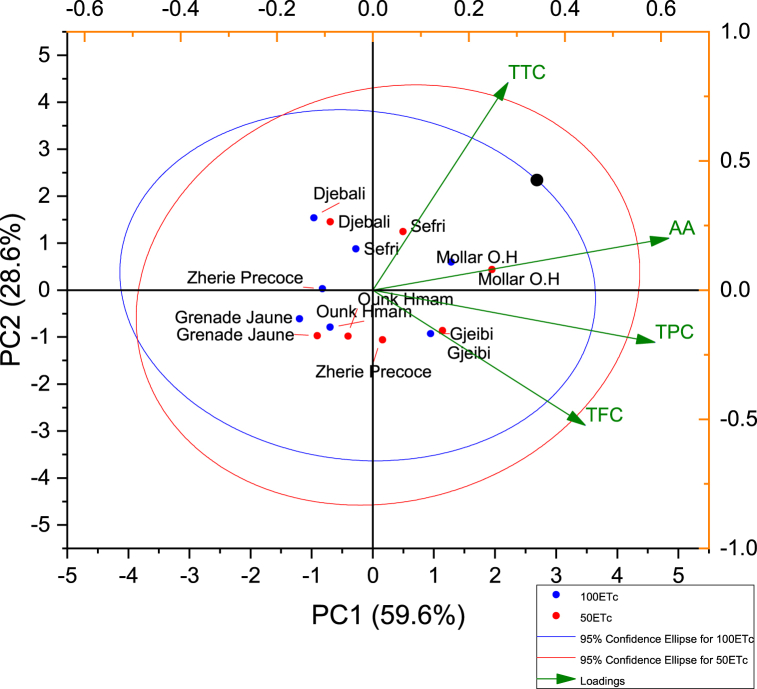
Biplot based on biochemical traits of samples pomegranates seeds oil as affected by water stress. Loading of biochemical variables on each principal component were plotted in green color.

**Fig. 7:**
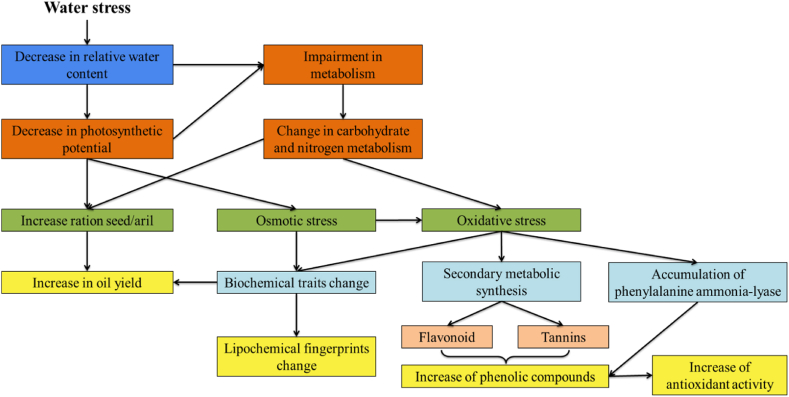
Overall mechanisms triggered in pomegranate seeds as a response to drought highlighting the complex network of physiological and biochemical processes involved in the species' drought resilience.

In terms of biochemical properties, the observed increase in total phenolic content and antioxidant activity under water stress conditions can likely be attributed to the role of phenolic compounds as secondary metabolites involved in plant stress responses. Phenolic compounds have been shown to have antioxidant and anti-inflammatory properties, and their production can increase in response to various types of stress, including water stress.

Furthermore, the observed changes in lipochemical fingerprints under water stress conditions can be attributed to alterations in the composition of the fatty acids present in the oil. Specifically, water stress appears to increase the proportion of certain fatty acids, such as oleic acid and linoleic acid, which are associated with improved oil quality and health benefits. This shift in fatty acid composition may be due to alterations in the activity of enzymes involved in lipid metabolism, or changes in the expression of genes involved in lipid biosynthesis.

Overall, these mechanisms suggest that pomegranate plants have a degree of resilience to water stress conditions, and that this resilience is reflected in changes in oil yield, biochemical properties, and lipochemical fingerprints. Further research is needed to fully understand the mechanisms underlying these changes and to develop strategies for improving pomegranate cultivation under water-limited conditions.

### 4.5 Multivariate analysis

**Fig. 8:**
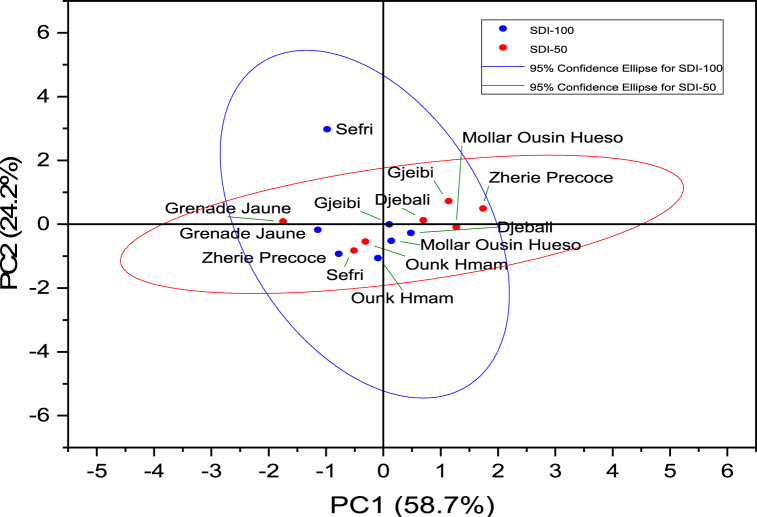
Bilplot based on FTIR data of all sampled seeds oil as affected by water stress (total variance = 82.9%).

Principal component analysis (PCA) based on correlation coefficients was performed to assess the cultivars herein examined to water stress and the variables most involved in their behavior. Two PCA models were separately built based on bio-lipochemical attributes and FTIR data to ascertain whether both approaches can provide a similar throughput resolution level. Fig. 8 is a biplot of PAC built based on lipobiochemical attributes, which accounted for 88.2% of the total variance explained by the two first components. Hence, the first component (PC1) represents more than 59.6% of the total variance, while the second component (PC2) accounted for 28.6% of the total variance and is mainly correlated with the recorded effects of applied water stress on the total phenolic content and antioxidant activity, which recorded the highest discriminating effect of SDI-50 on the seeds oil of the seven pomegranate cultivars involved in this study.

The biplot displayed that the response to water stress is almost similar among all analyzed cultivars. Indeed, similar regrouping of cultivars was seen both under full irrigation and water stress treatments. However, the analysis of the response of each cultivar to water stress showed a specific resilience of ‘Sefri’ and ‘Zheri Precoce’ to water stresses (Fig. 6). These results were explained by the fact that the applied water stress induced a significant increase in the total phenols, flavonoids and antioxidant activity content of the oil seeds of both cultivars.

**Fig. 9:**
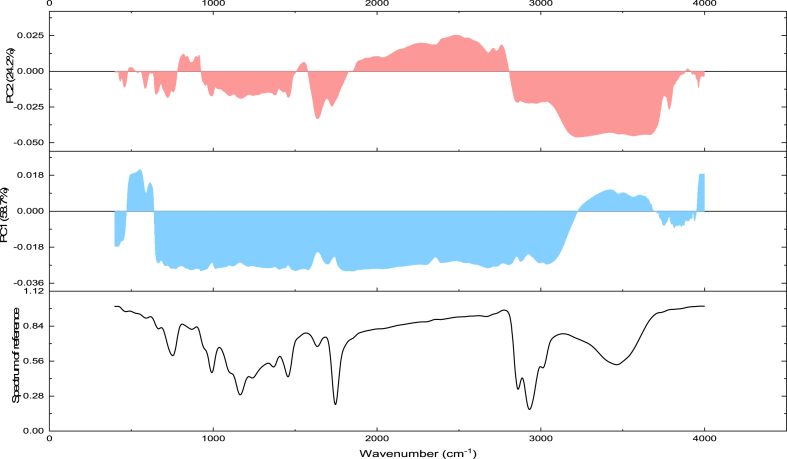
Loadings of each wavelength number in the PCA model total variance for the investigated pomegranate seeds oil samples.

FTIR-based PCA model displayed a cumulative variance of 82.9% explained by the two first components accounting for each 58.7% and 24.2%, respectively (Fig. 8, Fig. 9). The first component was mainly explained by the fingerprints at the vibrational region between 500 and 3000 cm^−1^ overall ascribed to triacyglycerols and proteins. The second is mainly correlated to the amide functions vibrations. All herein studied cultivars were similarly classified under full and deficit irrigation conditions except the ‘Sefri’ cultivar, as it was classified with discrimination to the water regime. This variation was mainly distinguished by a characteristic peak at 3471 cm^−1^ vibrational regions ascribed to NH stretch, coupled with HB.

## 5 Conclusion

This study sheds light on the potential for investigating the effects of drought by examining the lipo-biochemical attributes of pomegranate seeds, thereby allowing for an assessment of whether drought can influence the prospects of shifting to a circular economy model in the pomegranate processing pathway. In particular, the simulation of increased oil and phenolics production induced by drought presents a tangible opportunity to tackle the ongoing climate change crisis through the use of resilient cultivars under controlled deficit irrigation. The research also identified significant variations in the oil yield and biochemical attributes of the studied pomegranate cultivars in response to water stress. Specifically, water stress was found to enhance the lipo-biochemical quality of oil and increase its yield. Moreover, most cultivars demonstrated an increase in total phenolics content under water stress (SDI-50), thereby resulting in improved antioxidant capacity. Multivariate analysis revealed that ‘Sefri’ and ‘Zheri Precoce’ cultivars exhibited the most substantial changes in their oil biochemical traits relative to the studied collection. In contrast, FTIR data highlighted ‘Sefri’ as the most responsive cultivar to water stress, as evidenced by the heightened absorption intensity around the peak at 3471 cm^−1^, attributed to NH stretch, coupled with HB. Nonetheless, further research is necessary to fully leverage the potential of pomegranate seeds fingerprints to investigate pomegranate resilience under water deficit conditions and to seek other techniques for increasing the yield of the pomegranate seeds oil and to improve their lipochemical properties by exploiting other environmental conditions such as heat and salt stress.

## Author contribution statement

Atman Adiba: Formal analysis, Data curation, Writing – original draft.

Lahcen Hssaini: Funding acquisition, Formal analysis, Data curation, Writing–review & editing.

Abdelmajid Haddioui: Visualization. Rachida Ouaabou: Formal analysis, Visualization.

Anas Hamdani & Mohammed Kouighat: Visualization. Rachid Razouk: Conceptualization & Visualization.

## Declaration of competing interest

The authors declare that they have no known competing financial interests or personal relationships that could have appeared to influence the work reported in this paper.
